# Wine tannins: Where are they coming from? A method to access the importance of berry part on wine tannins content

**DOI:** 10.1016/j.mex.2020.100961

**Published:** 2020-06-12

**Authors:** Pauline Rousserie, Soizic Lacampagne, Sandra Vanbrabant, Amélie Rabot, Laurence Geny-Denis

**Affiliations:** Unité de Recherche Œnologie, Université de Bordeaux, EA 4577, USC 1366 INRA, ISVV, 33882 Villenave d'Ornon Cedex, France

**Keywords:** Winemaking, Tannins, Seeds, Skins, *Vitis vinifera*

## Abstract

Phenolic compounds are important constituents of red wine involved in its sensory properties. Although wine tannins can come from microbial and oak sources, the main sources of polyphenol remains grape skins and seeds. In order to better understand the grape seed and skin tannins contribution to the final wine tannins content, an original approach of winemaking has been set up. Our protocol explains a simple method to determine the percentage of skin and seed tannins extracted in wine all along the winemaking. The advantages of this method are presented below:

• This method allows us to describe tannins extraction kinetics in wine according to the berry part (skins and seeds).
• Additionally, our protocol allows to specifically determine the percentage of tannins extraction according to the winemaking stages (alcoholic fermentation and post-fermentative maceration) and the berry part (skins and seeds).
• To sum up, this method serves to enhance the comprehension of tannins extraction process in wine according to the berry part.

## Graphical abstract

**Figure:**
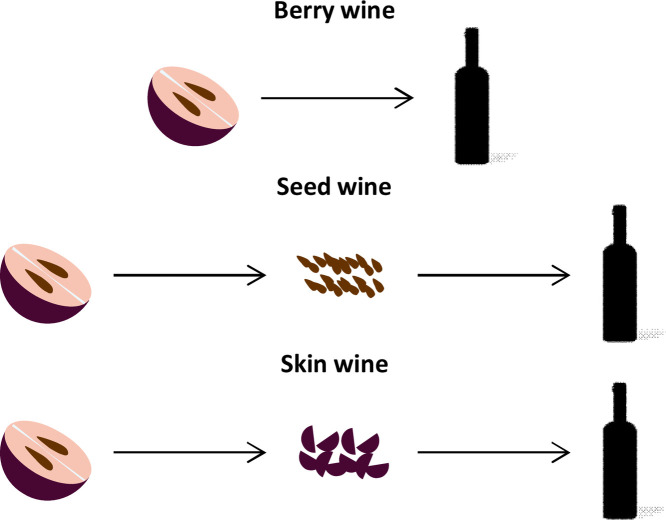

Specifications table

**Table:** 

Subject area:	Agricultural and Biological Sciences
More specific subject area:	Œnology
Method name:	Specific nanovinification to determinate the origin of wine tannins
Name and reference of original method:	The method developed here in inspired from: Sparrow, A. M., Dambergs, R. G., Bindon, K. A., Smith, P. A., & Close, D. C. (2015). Interaction of grape skin, seed, and pulp on tannin and anthocyanin extraction in Pinot noir wines. American Journal of Enology and Viticulture, 66(4), 472–481. https://doi.org/10.5344/ajev.2015.15022
Resource availability:	NA

## Method details

### Overview

Tannins and anthocyanins are the principal phenolic compounds of wine, conferring upon red wines their particular organoleptic properties. Tannins contribute to the mouthfeel of wines but they also form pigmented polymers in association with the anthocyanins to provide the stable pigments required to give red wine its long-term color stability [3]. In grape, the total of extractable phenolics are distributed as follows: 10% or less in pulp, 28% to 35% in skins and 60% to 70% in seeds [5]. Even though some studies have estimated that 9% to 50% of grape tannins are incorporated in wine, a relatively poor number of published studies deal with the link between grape phenolic and wine composition [[1], [2], [3],^7^]. Furthermore, because of the disruption of tissues (destemming, crushing) prior to vinification, the relative contribution of anatomically distinct berry tissues (skins, seeds and pulp) to wine tannins remains difficult to establish.

To our knowledge only one method exist to determine the percentage of seed and skin tannins extraction in red wine [4]. This analytical method is based on the analysis of wine proanthocyanidins cleavage products after acid catalysis in the presence of excess phloroglucinol. This method is suitable to estimate the percentage of seed tannins and skin tannins that are present in wine, but it does not permits to specifically know the percentage of seed and skin tannins that are released in wine. In this method, after having characterized the tannins content of fresh seed and skin, we propose to analyze grape seed and grape skin tannins content all along the vinification. By doing so, the percentage of tannins release in wine by seeds and skins can be calculated.

### Required reagents and equipment

• One-liter fermenter
• Saccharomyces cerevisiae
• Sulfite solution
• Densitometer
• Dry ice
• Grape berries

### Procedure

#### Berries

Approximatively 20 kg of berries are needed to conduct the experiment

Among the 20 kg of berries, one sample of five hundred berries was taken to evaluate ripening level and berries characteristics. Three groups of one hundred berries were destemmed and crushed to estimate the volume of juice and the parameters of maturity level: total soluble solids (TS) (° Brix), potential alcohol content, titrable acidity (g L^−1^), pH value, tartaric acid (g L^−1^) and malic acid (g L^−1^) were measured with a WineScan™ Flex (Foss, Hilleroed, Denmark) coupled to Foss Integrator 2 software (version 2.0.2). The other berries were used to estimate the berry weight, the seed number by berry and the seed weight.

At the end of this part of the method the following information are collected:

• The average weight of one berry
• The average weight of one seed
• The average number of seeds per berry
• The average weight of one skin
• The average volume of juice of 100 berries.

#### Winemaking

**Fig. 1:**
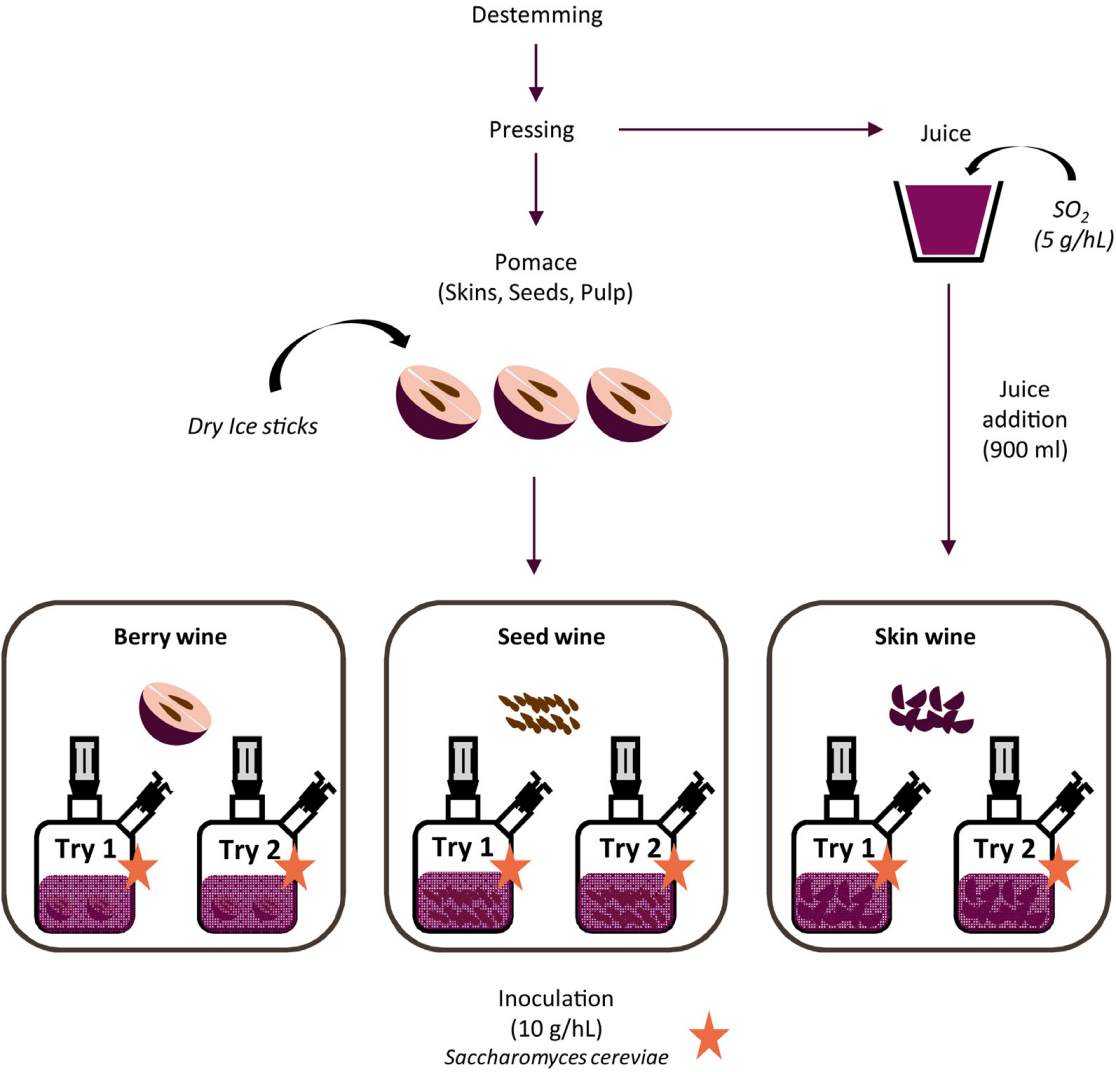
Winemaking method.

Wines were elaborated at Plateau de Nanovinification de l'unité de recherché oenologie of the Institut des Sciences de la Vigne et du Vin (University of Bordeaux). In order to evaluate the effect of each berry tissue component in wine, three winemaking modalities have been produced in duplicate: a modality control called berry wine, a modality seed called seed wine and a modality skin called skin wine (Fig. 1).

Grapes were destemmed and crushed in order to collect the grape juice. The grape juice was sulphited at 5 g hL^−1^ in order to avoid oxidation. After have been crushed, berries were collected and combined with dry ice sticks to avoid oxidation. Then, skins and seeds, always under dry ice sticks, were separated manually.

For berry modality, 900 ml of grape juice were mixed with the corresponding amount of berries known thanks to the volume of juice of 100 berries. For seed modality, 900 ml of grape juice were mixed with the corresponding amount of seed known thanks to the volume of juice of 100 berries, the average number of seed per berry and the average weight of one seed. For skin modality, 900 ml of grape juice were mixed with the corresponding amount of skin known thanks to the volume of juice from 100 berries and the average weight of one berry skin. Seed, skin and pomace weight for a volume of 900 ml have been calculated as follow:

$$\begin{array}{c} \text{Seed}\;\text{weight}\;\left(\text{in}\;\text{fermenter}\right)\;=\frac{100\;\times \;900}{\text{Juice}\;\text{volume}\;\text{of}\;100\;\text{berries}}\\ \times \;\text{Number}\;\text{of}\;\text{seed}\;\text{per}\;\text{berry}\;\times \;\text{Fresh}\;\text{seed}\;\text{weight} \end{array}$$

$$\text{Skin}\;\text{weight}\;\left(\text{in}\;\text{fermenter}\right)=\frac{100\;\times \;900}{\text{Juice}\;\text{volume}\;\text{of}\;100\;\text{berries}}\times \;\text{Fresh}\;\text{skin}\;\text{weight}$$

Pomaceweight=Seedweight+Skinweight

All experiments have been made into 1 L fermenters. Fermentations were performed with a *Saccharomyces cerevisiae* yeast inoculum of strain Actiflore F33 (Laffort, Bordeaux, France) at 10 g hL^−1^. All fermentations were conducted at 20 °C, and were monitored daily by measuring the density with a digital densitometer (Anton Paar, model DMA 35).

#### Sampling method

**Fig. 2:**
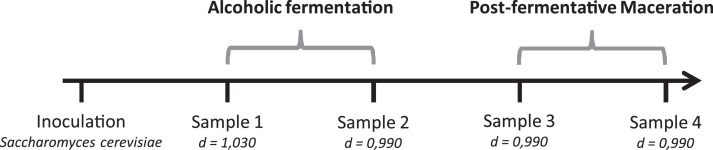
Sampling method.

During winemaking four samples were made on each modality (berry, seed and skin wine): one at half alcoholic fermentation (density = 1.030), one at the end of alcoholic fermentation (density = 0.990), one at the middle of the post-fermentative maceration (approximately one week after the end of the alcoholic fermentation) and one at the end of the post-fermentative maceration (approximately two weeks after the end of the alcoholic fermentation) (Fig. 2).

For each sample, 20 mL of wine were collected. In order to keep the ratio vegetal material:juice stable, at each sample, the correspondent amount of berries, seeds and skin were respectively removed from the berry modality, the seed modality and the skin modality. The mass of vegetal material taken from the fermenters has been calculated has followed:

$$\text{Vegetal}\;\text{material}\;\text{weight}\;=\frac{\text{Total}\;\text{amount}\;\text{of}\;\text{vegetal}\;\text{material}\;\text{in}\;\text{fermenter}}{900}\times \;20$$

#### Method validation

**Fig. 3:**
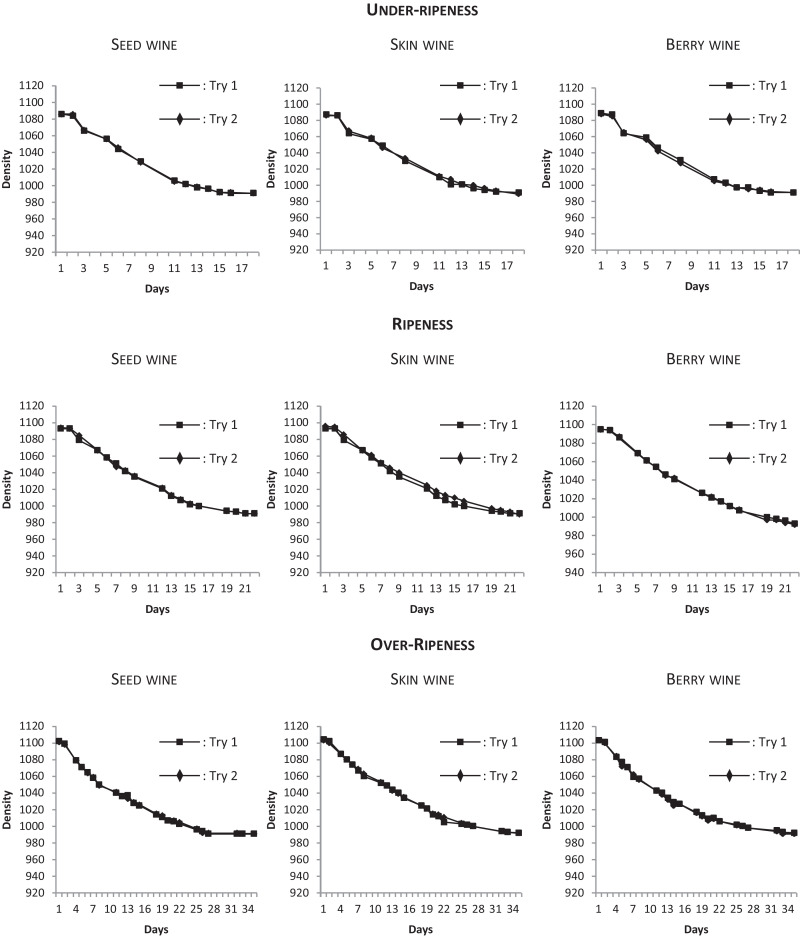
Fermentation kinetics for each type of wine at the three ripeness degrees.

To validate the method, the experiments have been conducted three times at three different maturity stages: under-ripeness, commercial ripeness and over-ripeness. The fermentation kinetics of all the experiments have been modeled. The results of density determinations during alcoholic fermentation for each modality are presented in Fig. 3.

For each type of wines (seed wine, skin wine and berry wine) the duplicate (try 1 and try 2) present the same fermentation kinetics. To go further, for each ripeness stages (under-ripeness, ripeness and over ripeness), fermentations of both seed, skin and berry wines are comparable:

• For the under-ripeness modality, for all the type of wines, fermentations were completed on 18 days;
• For the ripeness modality, for all the type of wines, fermentations were completed on 22 days;
• For the over-ripeness modality, for all the type of wines, fermentations were completed on 34 days.

The difference of alcoholic fermentation time between the three ripeness stages is related to the sugar composition of grape juice. Indeed, at the under-ripeness stage the sugar content was lower than the one of the over-ripeness stage.

To sum up, it seems that the winemaking and the sampling methods do not affect the successful completion of the alcoholic fermentation, which allows us to validate the method.

## Conclusion

This method is allowing the sampling of wine and pomace during winemaking without modifying the successful achievement of the alcoholic fermentation. Considering this fact, the vegetal material and wine collected at the different winemaking steps can be used to analyze the extraction and the kinetics extraction of compounds of interest, such as tannins, from pomace to wine all along the winemaking [6].

## Declaration of Competing Interest

The authors declare that they have no known competing financial interests or personal relationships that could have appeared to influence work reported in this paper.
